# Medical students can easily acquire intraosseous cannulation skills

**DOI:** 10.1186/1757-7241-23-S1-A46

**Published:** 2015-07-16

**Authors:** Ask D Kvisselgaard, Monika Afzali, Tobias S Lyngeraa, Sandra R Viggers

**Affiliations:** 1 Students' Society for Anaesthesiology and Traumatology, Faculty of Health Sciences, Copenhagen University, Copenhagen, Denmark; 2 Department of Anaesthesiology, Bispebjerg Hospital, Copenhagen, Denmark

## Background

In life-threatening emergencies with intravascular volume depletion, shock, or even cardiac arrest, obtaining conventional intravascular access can be difficult due to peripheral vascular shutdown. Intraosseous (IO) access is recommended during resuscitation when conventional intravenous access is difficult and is a fast and safe method for administration of fluids, blood products, and medications. However, lack of training in the procedure may be a reason why the use of the IO access is still limited in resuscitation. The goal of this study was to investigate if medical students can obtain competencies in IO access taught on a human cadaver course.

## Methods

A total of 19 medical students (4^th^-12^th ^semester) from the University of Copenhagen, all members of the Students' Society of Anaesthesiology and Traumatology, participated in the course. A modified Peyton's four-step approach was used for the hands-on training preceded by a short theoretical lesson. Following the course, three observers evaluated performance during a procedure-specific objective structured clinical examination (OSCE). The OSCE checklist was developed by the authors from existing guidelines and previous literature. Inter-observer agreement with Randolph's free-marginal multirater *kappa *was compared to evaluate validity of the checklist.

## Results

In the final OSCE, 15 students participated. A total of 11 students (73%) obtained the highest attainable points; 15. The median total score was 15 (12-15). There was no correlation between the failed items on the checklist for the four students who did not receive maximal points.

The free-marginal *kappa *value was calculated to 0.7066 indicating substantial agreement between the observers.

## Conclusion

This study has demonstrated that the fundamentals of safe IO access can be taught to medical students through a human cadaver course. Further studies are needed to validate the retention of the gained knowledge and different teaching modalities should be tested against each other. We suggest that the training in IO access could be a part of the curriculum in medical school to ensure the highest standard of care in resuscitation.

